# Evidence for Limited Early Spread of COVID-19 Within the United States, January–February 2020

**DOI:** 10.15585/mmwr.mm6922e1

**Published:** 2020-06-05

**Authors:** CDC COVID-19 Response Team, Michelle A. Jorden, Sarah L. Rudman, Elsa Villarino, Stacey Hoferka, Megan T. Patel, Kelley Bemis, Cristal R. Simmons, Megan Jespersen, Jenna Iberg Johnson, Elizabeth Mytty, Katherine D. Arends, Justin J. Henderson, Robert W. Mathes, Charlene X. Weng, Jeffrey Duchin, Jennifer Lenahan, Natasha Close, Trevor Bedford, Michael Boeckh, Helen Y. Chu, Janet A. Englund, Michael Famulare, Deborah A. Nickerson, Mark J. Rieder, Jay Shendure, Lea M. Starita, CDC COVID-19 Response Team, Gregory L. Armstrong, Jay C. Butler, Michael A. Coletta, Aaron Kite-Powell, Julu Bhatnagar, Sarah Reagan-Steiner, Suxiang Tong, Brendan Flannery, Jill M. Ferdinands, Jessie R. Chung

**Affiliations:** ^1^County of Santa Clara Office of the Medical Examiner-Coroner, San Jose, California; ^2^County of Santa Clara Public Health Department, San Jose, California; ^3^Illinois Department of Public Health; ^4^Cook County Department of Public Health, Chicago, Illinois; ^5^Chicago Department of Public Health; ^6^Louisiana Office of Public Health; ^7^Michigan Department of Health and Human Services; ^8^New York City Department of Health and Mental Hygiene; ^9^New York State Department of Health; ^10^Public Health – Seattle & King County, Washington; ^11^Washington State Department of Health; ^12^Fred Hutchinson Cancer Research Center, Seattle, Washington; ^13^University of Washington, Seattle, Washington; ^14^Seattle Children's Hospital, Seattle, Washington; ^15^Institute for Disease Modeling, Bellevue, Washington; ^16^Brotman Baty Institute for Precision Medicine, University of Washington, Seattle, Washington.; CDC; CDC; CDC; CDC; CDC; CDC; CDC; CDC; CDC; CDC.

*On May 29, 2020, this report was posted online as an *MMWR *Early Release.*

From January 21 through February 23, 2020, public health agencies detected 14 U.S. cases of coronavirus disease 2019 (COVID-19), all related to travel from China (*1*,*2*). The first nontravel–related U.S. case was confirmed on February 26 in a California resident who had become ill on February 13 (*3*). Two days later, on February 28, a second nontravel–related case was confirmed in the state of Washington (*4*,*5*). Examination of four lines of evidence provides insight into the timing of introduction and early transmission of SARS-CoV-2, the virus that causes COVID-19, into the United States before the detection of these two cases. First, syndromic surveillance based on emergency department records from counties affected early by the pandemic did not show an increase in visits for COVID-19–like illness before February 28. Second, retrospective SARS-CoV-2 testing of approximately 11,000 respiratory specimens from several U.S. locations beginning January 1 identified no positive results before February 20. Third, analysis of viral RNA sequences from early cases suggested that a single lineage of virus imported directly or indirectly from China began circulating in the United States between January 18 and February 9, followed by several SARS-CoV-2 importations from Europe. Finally, the occurrence of three cases, one in a California resident who died on February 6, a second in another resident of the same county who died February 17, and a third in an unidentified passenger or crew member aboard a Pacific cruise ship that left San Francisco on February 11, confirms cryptic circulation of the virus by early February. These data indicate that sustained, community transmission had begun before detection of the first two nontravel–related U.S. cases, likely resulting from the importation of a single lineage of virus from China in late January or early February, followed by several importations from Europe. The widespread emergence of COVID-19 throughout the United States after February highlights the importance of robust public health systems to respond rapidly to emerging infectious threats.

## Syndromic Surveillance

Through the National Syndromic Surveillance Program, U.S. public health agencies receive real-time data from emergency departments in approximately 4,000 health care facilities in 47 U.S. states and the District of Columbia. In 14 counties with early community-acquired cases of COVID-19, no substantial increase was observed in the proportion of COVID-19–like illness (fever and cough or shortness of breath or difficulty breathing, or the listing of a coronavirus diagnostic code) before February 28 (Figure).

**FIGURE:**
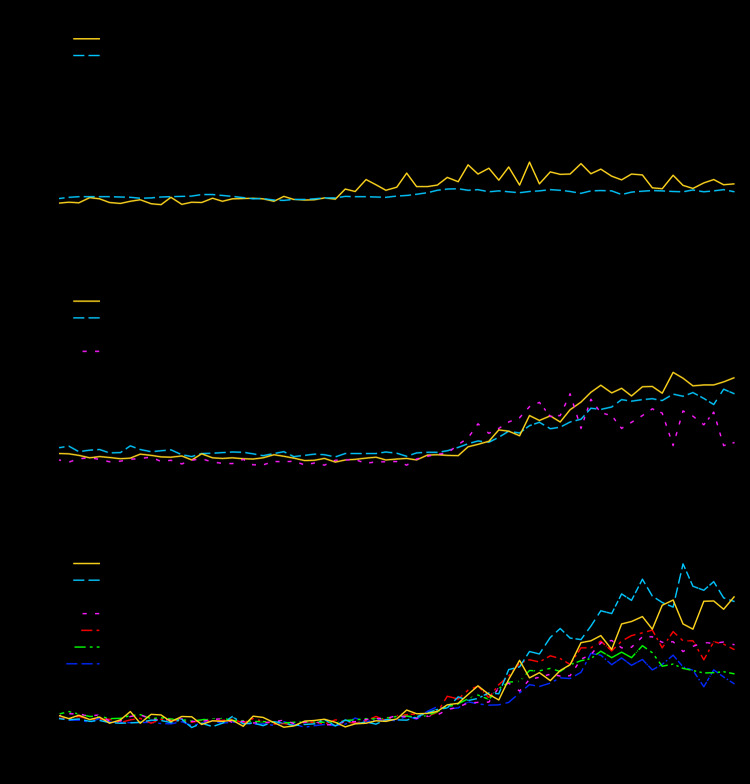
Percentage of emergency department (ED) visits for COVID-19–like illness (CLI),* in 14 counties^†^^,^^§^ (three in California and Washington [A]; four in Illinois, Louisiana, Massachusetts, and Michigan [B]; and seven in New York [C]) — National Syndromic Surveillance System,^¶^ February 1–April 7, 2020 **Abbreviation:** COVID-19 = coronavirus disease 2019. * Fever and cough or shortness of breath or difficulty breathing or presence of a coronavirus diagnostic code. ^†^ *California:* Santa Clara County; *Washington:* King County, Snohomish County; *Illinois:* Cook County; *Louisiana:* Orleans Parish; *Massachusetts:* Middlesex County; *Michigan:* Wayne County; *New York:* Bronx County, Kings County, Nassau County, New York County, Richmond County, Queens County, Westchester County. ^§^ King County, Washington includes Seattle; Cook County, Illinois includes Chicago and many of its suburbs; Wayne County, Michigan includes Detroit and many of its suburbs; Orleans Parish includes New Orleans; Kings County (Brooklyn), Queens County (Queens), Bronx County (Bronx), Richmond County (Staten Island), and New York County (Manhattan) are all within New York City. ^¶^ From the subset of emergency departments in each county that participates in the National Syndromic Surveillance Program.

## Surveillance for Acute SARS-CoV-2 Infection

The Seattle Flu Study (*5*) began monitoring acute respiratory disease in the Seattle metropolitan area in November 2018. In late February 2020, the study began testing specimens using reverse transcription–polymerase chain reaction (RT-PCR) testing for SARS-CoV-2. The first positive laboratory result for SARS-CoV-2 was detected on February 28 from a specimen collected February 24. After this detection, deidentified specimens collected earlier were retrospectively tested for the virus. There were no positive results among 5,270 respiratory specimens collected during January 1–February 20 (*5*) (T. Bedford, Fred Hutchinson Cancer Research Center, Seattle, Washington, personal communication, May 6, 2020).

The first specimen that tested positive among these retrospectively tested specimens had been collected February 21. During the week beginning February 21, eight of 1,255 specimens (0.6%) tested positive, and during the following week, 29 of 1,862 (1.6%) specimens tested positive.

Two influenza vaccine effectiveness study networks with sites in six states (Michigan, Pennsylvania, Tennessee, Texas, Washington, and Wisconsin)* retrospectively tested respiratory specimens from patients with acute respiratory disease for SARS-CoV-2 by RT-PCR. At the Washington site, none of the 497 specimens collected during January 19–February 24 tested positive; the first specimen that tested positive was collected on February 25. At the five other sites (Ann Arbor and Detroit, Michigan; Pittsburgh, Pennsylvania; Temple, Texas; Marshfield, Wisconsin; and Nashville, Tennessee), none of 2,620 samples collected during January 19–February 29 tested positive for SARS-CoV-2.

As of May 22, 2020, four (<0.2%) of approximately 3,000 specimens collected from children and adolescents aged <18 years enrolled in the New Vaccine Surveillance Network^†^ during January 1–March 31 have tested positive for SARS-CoV-2. The earliest positive result was from a specimen collected March 20 in Seattle.

## Phylogenetic Analysis

Analysis of the genomic diversity of SARS-CoV-2 from early cases of COVID-19 from the Seattle area found that most viruses belonged to a single clade (the Washington State clade), whose most recent common ancestor was estimated to have existed between approximately January 18 and February 9 (point estimate = February 1).^§^ The predicted genomic sequence of that progenitor virus was consistent with that from the first U.S. case of imported COVID-19, which occurred in a man who arrived in Seattle from Wuhan, China, on January 15 and became ill 4 days later. However, it is also possible that the Washington State clade arose from a virus with a similar or identical sequence from another person with SARS-CoV-2 infection. Analysis of viruses in California and the northeastern United States from February through mid-March suggested that there had been several importations of virus, primarily from Europe, followed by transmission of virus within the United States^¶^^,^** (*6*).

## Known Cases in Persons with No Relevant Travel History Before February 26

Two notable cases of COVID-19 occurred in Santa Clara County, California: one in a woman who became ill on January 31 and died on February 6 and another in an unrelated man who died at home between February 13 and 17. Neither had traveled internationally in the weeks preceding their deaths. SARS-CoV-2 RNA was detected by RT-PCR testing at CDC from postmortem tissue specimens from these patients. These deaths were certified by a medical examiner as COVID-19–associated deaths. Investigation of these cases is ongoing.

Outbreaks of COVID-19 occurred during two consecutive voyages of a Grand Princess cruise ship (*7*). The genomic sequence of viruses from these outbreaks was within the Washington State clade, suggesting that a passenger or crew member infected with that virus was aboard the ship when it left the Port of San Francisco on February 11 for a round-trip cruise. The identity of that person is unknown.

## Discussion

Information from these diverse data sources suggests that limited community transmission of SARS-CoV-2 in the United States occurred between the latter half of January and the beginning of February, following an importation of SARS-CoV-2 from China. This importation initiated a lineage, the Washington State clade, which subsequently spread throughout the Seattle metropolitan area and possibly elsewhere. Several importations of SARS-CoV-2 from Europe followed in February and March. It is not known how many U.S. infections occurred during February and March, but overall disease incidence before February 28 was too low to be detected through emergency department syndromic surveillance data.

Also unknown are the dates of entry of the imported viruses into the United States and the identities of the persons who carried them. One possible early source is the first reported U.S. case of COVID-19, which occurred in a Washington man who became ill on January 19 after returning from Wuhan, China, on January 15; the genomic sequence of the virus isolated from that man is consistent with his being the possible source of the Washington State clade, although the thoroughness of the contact investigation of this case and the absence of identified secondary cases argue against this (*8*). However, subsequent published reports have indicated that infection with SARS-CoV-2 is frequently asymptomatic and that transmission can occur before the onset of symptoms (*9*). The possibility of presymptomatic transmission raises at least three other potential scenarios involving this case: 1) that one or more secondary asymptomatic infections might have occurred among the patient’s contacts and that these led to further, undetected spread of the virus; 2) that the man might have infected contacts before his symptom onset (such contacts would not have been identified through the standard recommended contact investigation at that time); or 3) that he and at least one other person were infected by another passenger on the same flight from Wuhan, and undetected spread from the other infected persons gave rise to the Washington State clade. Which, if any, of these scenarios occurred likely will never be known. It is also possible, given the limited global phylogenetic diversity of SARS-CoV-2 at the time, that the Washington State clade was imported into the United States by another, unknown person around the same time.

Results of serologic testing are not presented here, because serology (i.e., testing for antibody to SARS-CoV-2) is likely to be a relatively insensitive means of detecting a newly emergent virus, particularly when the specimens were collected at random rather than from persons most likely to be infected (in contrast, for example, to viral testing of outpatients or hospitalized patients with acute respiratory disease) and because serologic assays generally do not approach 100% specificity unless some form of confirmatory testing is available. For example, a hypothetical serologic survey in the Seattle metropolitan area (population of 3.5 million) conducted after the first 3,500 infections would find a true seroprevalence of 0.1%, whereas the use of an assay with 99% specificity would be expected to produce false positives in 10 times as many samples. Serologic surveys, nonetheless, are useful in tracking the progress of the pandemic once established and have the potential advantage of detecting all infections, regardless of the symptom profile.

The findings in this report are subject to at least three limitations. First, the data presented here are retrospective. Although they are geographically diverse, they cannot provide as definitive a picture of transmission as would be available had widespread testing been immediately available after discovery of the virus. Second, some of the studies cited and possibly others are continuing to test samples retrospectively and might find earlier cases than those presented in this report. Finally, the relative phylogenetic homogeneity of SARS-CoV-2 globally in January and early February limited what could be inferred from genomic analysis.

Few countries have avoided the importation and sustained spread of COVID-19. In the United States, SARS-CoV-2 is now circulating widely after several importations from China, Europe, and elsewhere. Steps are underway throughout the U.S. public health system to improve indicators of SARS-CoV-2 activity, including expanding syndromic surveillance among emergency departments and increasing the availability of testing for SARS-CoV-2. Given the probability that most of the U.S. population is still susceptible, sustained efforts to slow the spread of the virus are crucial, including effective contact tracing and nonpharmaceutical interventions, such as physical distancing and source control (i.e., wearing cloth face coverings).

**Summary**

**What is already known about this topic?**

The first U.S. cases of nontravel–related COVID-19 were confirmed on February 26 and 28, 2020, suggesting that community transmission was occurring by late February.

**What is added by the report?**

Four separate lines of evidence (syndromic surveillance, virus surveillance, phylogenetic analysis, and retrospectively identified cases) suggest that limited U.S. community transmission likely began in late January or early February 2020, after a single importation from China, followed by multiple importations from Europe. Until late February, COVID-19 incidence was too low to be detected by emergency department syndromic surveillance for COVID-19–like illness.

**What are the implications for public health practice?**

Enhanced syndromic and virus surveillance will be needed to monitor COVID-19 trends for the duration of the pandemic.

## Footnotes

* The U.S. Influenza Vaccine Effectiveness Network (five study sites, including sites in Michigan, Pennsylvania, Texas, Washington, and Wisconsin) and Hospitalized Adult Influenza Vaccine Effectiveness Network (includes sites in Michigan, Pennsylvania, Tennessee, and Texas).

† Rochester, New York; Pittsburgh, Pennsylvania; Cincinnati, Ohio; Nashville, Tennessee; Kansas City, Missouri; Houston, Texas; and Seattle, Washington.

§ https://www.medrxiv.org/content/10.1101/2020.04.02.20051417v2.

¶ https://www.medrxiv.org/content/10.1101/2020.04.08.20056929v2.

** https://www.medrxiv.org/content/10.1101/2020.03.27.20044925v1.
